# Cursory Observations on the Importance of Anatomical Knowledge in the Treatment of Diseases, with Remarks on Two Cases of Hydrocephalus Internus

**Published:** 1799-09

**Authors:** Charles Brown

**Affiliations:** Surgeon, Member of the Corporation of Surgeons of London


### *Cursory Observations on the Importance of anatomical Knowledge in the Treatment of Diseases, with Remarks on two Cases of Hydrocephalus internus.*—By CHARLES BROWN, *Surgeon, Member of the Corporation of Surgeons of London.*

*To the Editors of the Medical and Physical Journal.*

GENTLEMEN,

MEDICAL and chirurgical practitioners, from the time of HIPPOCRATES to the present day, have ever considered the study of anatomy, as forming one of the most essential branches of their education, and without which, they were not qualified to practise the *ars medendi*. Independent of its being one of the most entertaining, it is also the most useful part of natural philosophy. It expands our ideas, opens the understanding, and tends strongly to erase prejudice from the mind.

The zeal with which it is now cultivated, the large sums of money which have been expended in anatomical museums, and the priority and boldness which a surgeon, skilled in anatomy, assumes in the performance of any capital operation, are convincing proofs of its great utility.

One of the most able anatomists and physiologists of the present day (Mr. JOHN BELL), and one whose writings bear sufficient proof of this assertion, observes, "that a consciousness of the high value of anatomical knowledge never entirely leaves the mind of the student."

We are indebted to the illustrious MORGAGNI for his valuable work, "*De causis et sedibus morborum,*" a work abounding with many excellent practical remarks, but more so for that part which more immediately relates to, and is connected with *morbid anatomy*. Doctor BAILIE has also added a rich a rich ftore to our anatomical knowledge, by the publication of his invaluable work, entitled, "*The morbid Anatomy of fome of the moft important parts of the Human Body.*" The number of valuable cafes to be found in fome of our periodical publications, have alfo affifted the ftudent in his inquiries after difeafed appearances. Having premifed thefe few obfervations on the importance of cultivating this branch of fcience, I would at the fame time infift on the propriety of every medical man, ufing his utmoft exertions in overcoming the fuperftition and prejudice which attach to the minds of fome perfons, who contumacioufly refufe the bodies of their deceafed friends being examined, although it were to lead to fome great improvement in the practice of phyfic.

I purpofe therefore, from time to time, relating with the utmoft exactnefs, any morbid appearances, which I can obtain leave to examine in my own practice, or that of other gentlemen, and accompanying thofe remarks with further obfervations on the practice purfued in the treatment of fuch difeafes which lead to the inveftigation.

I have the honour to be

Your's obliged,

No. 25, Hatton-Garden. CHARLES BROWN.

*Two Cafes of Hydrocephalus internus, with practical Remarks.*

It is the opinion of all enlightened philofophers of the prefent day, that in order to get at truth, and accumulate ufeful facts (which, particularly in medicine, are indifputably much wanted), we ought to fufpect our own pre-conceived opinions, to diveft ourfelves as much as may be of our prejudices, and make ufe of the greateft impartiality in judging. If we look around us, we muft obferve, even among our moft intimate friends, how greatly they are biaffed to the ideas which have been engrafted on their underftanding, by education and cuftom; notwithftanding fome of them may involve the greateft abfurdities:—Thus we find, for inftance, many of the wives of the Indian kings are brought to believe, that by felf-murder (a crime which we think the greateft and moft contrary to nature) they fhall inherit the greateft blifs.—Under this perfuafion, many throw themfelves into the funeral pile with their dead hufbands, encouraged and animated to it, by thofe who are believed to be the beft and wifeft of their people. We may alfo obferve, that a perfon educated in Italy will be a Catholic; in England, a Proteftant; in Turkey, a Mahometan; in India, a Gentoo:—Errors from the fame caufe reign in philofophy, as well as divinity and medicine; our philofophical opinions are generally formed in the fame manner, by education, and the company company we keep; and often under the protection of fome great name, whofe notions few dare to oppofe, or even to inveftigate and examine:—thus the earth, moon, and planets, were believed to circulate round the fun, by the ancients down to PYTHAGORAS. After that, the *Ptolemean* fyftem took place, then the *Tychonic*, and now the ancient fyftem is again recovered by COPERNICUS and his followers, and demonftrated to be true by the immortal NEWTON, and now univerfally believed.

The foregoing obfervations fhew us how eafily we are induced to believe falfe opinions by education, and with what difficulty they are eradicated afterwards.

I have been led to preface the following cafes with thefe remarks, with a view of drawing the attention of my readers to a difeafe of very confiderable importance, and which feldom yields to thofe remedies ufually employed.

There are fome difeafes, fays an elegant writer, "whofe fymptoms are fo obfcure, or fo fimilar to thofe of other difeafes, as to prevent us from forming a certain diagnoftic, till death enfues, and diffection afcertains the caufe." Of this number we may include *hydrocephalus internus;* for although this fatal complaint is accurately defcribed by late writers, yet there are few of the fymptoms attending it, as pathognomonic, but may arife from very different caufes: as preffure upon the brain, from various caufes, dentition, and even from worms in the inteftinal canal, according to the authority of medical writers. In a late publication, * I have endeavoured to lay down the diftinguifhing characteriftic between this difeafe and others which are ufhered in with the fame fymptoms; but moft diforders of children are, more or lefs, accompanied with anomalous appearances, particularly as to the ftate of the pupils, heavinefs about the eyes, picking of the nofe, and inactivity. In regard to the ufe of mercury in this difeafe, little dependence is to be placed upon it, nor is it ever exhibited in private practice, without being conjoined with opium, and very frequently the fol. digit. pur. Blifters are generally applied at the time the patient is under the mercurial courfe, as alfo leeches, electricity, and other topical remedies; fo that we can never be accurate in our commendation of any remedy which is employed in conjunction with others equally active, and poffeffing fimilar virtues. The two following cafes occurred nearly within a few days of each other; the firft was treated with mercurials, in confultation with an apothecary in my neighbourhood, and died; the other I had

* BROWN on Scrophulous Diseases, octavo.

I had the sole charge of myself, was treated in a contrary way, and recovered.

Case I. S. D. a child two years old, was attacked, June 5, 1799, with symptoms of hydrocephalus. The first suspicion of disease was indicated by great stupor, pain in the head, a distortion of the eyes, and a pulse beating 160 strokes in a minute. The child had long been troubled with worms, for which a patent vermifuge had been exhibited without success. The gentleman who first attended prescribed calomel and rhubarb, in small doses; but on the 12th, the pupils of the eyes became dilated, and insensible of light. *Strabismus* succeeded, and in the evening there were evident symptoms of apparent dissolution. Upon being called in, I ordered the head to be shaved, and blisters to be applied in the direction of the sutures. The following medicines were prescribed:

℞ Hydrargyr. muriat. mitis, gr. i. Opii. crud. pulv. gr. ss. Cretæ præp. gr. iij. M. fiat pulvis horâ somni exhibendus, et quartis horis repetendus.

Inung. nucha unguent. hydrarg. scrup. ij. bis quotidie.

June 13. The calomel has not proved purgative, and the patient slept last night, but was frequently troubled with convulsive motions of the arms and eyes, and tosses the head from one side to the other on the pillow. The pupils continue in the same state. Pulse 120.

Applicentur hirud. duæ, capiti.

Persistat in usû medicam. ut heri præscript.

June 14. Restless all night, vomited twice. Pulse this morning 122. Had two stools; very feverish; frequently screams out, as if in great pain; the pupils not so much enlarged. At nine o'clock this evening the muscles of the lower jaw were convulsed, the patient became very restless, and died apparently in great agony at twelve o'clock.

The next morning I opened the head, and discovered the lateral ventricles filled with water. The *cerebrum* was largely distended, in consequence of the *septum lucidum* having been ruptured, the two ventricles having then a free communication. One very remarkable circumstance shewed itself in this case, which was that the *infundibulum* was hollow, and filled with a diaphanous fluid. This is worthy of remark, as anatomists in general deny this being the case, although some late experiments on this part of the brain * by Professor Murray, of Upsal, clearly prove it to be a medullary canal,

* Disp. de infundibulo cerebri.

canal, surrounded by a lamina of the *pia mater*. He froze the brain, and found the canal leading to the *glandula pituitaria* filled with ice. De Haen * tells us, he found it dilated, and filled with a calcareous matter.

Case II. The patient was eighteen months old, and of a strumous habit, and had all the usual symptoms of the disease. I applied leeches to the temples, blisters to the head and nape of the neck; gave the fol. digit and calx zinci combined; when comatose, stimulated with sal. c. c. vol & æther: with a small electrifying machine, I passed a few shocks twice a day through the head. Gave opium to the amount of three grains each night *ad tres vices*. In nine days he was quite recovered. Hydrocephalus is evidently a disease of debility, and requires remedies which increase the excitability of the system. On a future occasion, I shall continue my remarks on this disorder.

*July* 5, 1799.

